# Implications of low-density microfilariae carriers in *Anopheles* transmission areas: molecular forms of *Anopheles gambiae* and *Anopheles funestus* populations in perspective

**DOI:** 10.1186/1756-3305-7-157

**Published:** 2014-04-01

**Authors:** Bethel Kwansa-Bentum, Fred Aboagye-Antwi, Joseph Otchere, Michael David Wilson, Daniel Adjei Boakye

**Affiliations:** 1 Department of Animal Biology and Conservation Science, University of Ghana, P.O. Box LG 67 Legon, Accra, Ghana; 2 Parasitology Department, Noguchi Memorial Institute for Medical Research, P.O. Box LG 581 Legon, Accra, Ghana

**Keywords:** Mass drug administration, Low-density microfilariae carriers, *Wuchereria bancrofti*, *Anopheles gambiae*, *Anopheles funestus*

## Abstract

**Background:**

Previous studies have shown a general reduction in annual transmission potential (ATP) of *Anopheles* species after mass drug administration (MDA) in lymphatic filariasis endemic communities. Whereas results obtained from a monitoring programme after three years of MDA revealed a decrease in ATP of *Anopheles funestus* this was not the same for *An. gambiae* s.s. in Ghana. In this study, the ability of these vectors in transmitting *Wuchereria bancrofti* in nine lymphatic filariasis endemic communities in Gomoa District of Ghana after four rounds of MDA with ivermectin and albendazole was investigated.

**Methods:**

After mass screening of inhabitants in these communities, twelve consenting volunteers with different intensities of microfilariae (mf) slept under partly opened mosquito nets as sources of mf blood meal. Hourly collection of mosquitoes and finger-pricked blood were taken from 21.00 to 06.00 hours the following day. For each hour, half of the mosquitoes collected were immediately killed and dissected for mf. The remaining half were maintained up to 13 days for parasite maturation. Parasitaemia and infection rates in the mosquitoes were determined by microscopy. The mosquitoes were identified by microscopy and molecular techniques.

**Results:**

A total of 1,083 participants were screened and the overall parasite prevalence was 1.6% with mf intensities ranging from 0 to 59 per 100 μl and geometric mean intensity of 1.1 mf per ml of blood. Of the 564 mosquitoes collected, 350 (62.1%) were *Anopheles* spp., from which 310 (88.6%) were *An. funestus* and 32 (9.1%) *An. gambiae*. Six anopheline mosquitoes (1.7%) were found infected with L_1_, but no larva was observed in any of the mosquitoes maintained up to 13 days. Molecular studies showed all *An. gambiae* s.l. to be *An. gambiae* s.s., of which 21 (70%) were of the M molecular form.

**Conclusion:**

At low-level parasitaemia after 4 rounds of MDA, there was no recovery of infective stage larvae of *W. bancrofti* in *An. funestus* s.l. as well as M and S forms of *An. gambiae*.

## Background

*Wuchereria bancrofti* is one of the three filarial worms responsible for about 90% of all lymphatic filariasis (LF) cases in the world [1]. These parasites are transmitted through the bite of infective mosquitoes of various genera. A competent vector is one that is capable of ingesting microfilariae (mf) from an infected human, supporting their development to the infective stage larvae (L_3_) and subsequently transmitting them to other uninfected persons. Depending on the vector species, its region of origin and the parasite density ingested, this ability to sustain the maturation and transmission of LF may be enhanced or restricted [2,3]. Low density mf is defined as the density of circulating mf in a specified blood compartment that cannot be detected in a significant number of instances when commonly used blood sampling techniques are applied in epidemiological studies; thus 4 mf per 20 μl (200 mf per ml) [4]. Zhang *et al.*[5] in their study reported that between 1.55 and 2.23% prevalence, there was a threshold provided no individual had an mf density of more than 12 mf per 60 μl of blood.

The Global Programme to Eliminate Lymphatic Filariasis (GPELF) was launched in 2000, with the main goal of halting transmission and reducing disability through annual mass drug administration (MDA) to all persons at risk of infection, particularly if the vectors are *Anopheles* species [6]. The strategy relies on the assumption that if the mf reservoir in the human host is reduced below a certain threshold, transmission of *W. bancrofti* by anopheline vectors could be interrupted [7]. This is due to the observation that even though *Anopheles* mosquitoes yield more infective stage larvae than *Culex* species, the latter is more efficient at ingesting and developing low-density mf (limitation) than the former [4]. Thus *Anopheles* mosquitoes are presumed to be efficient vectors of LF when the parasite density in the human population is high, a phenomenon known as “facilitation” [4]. This observation has been the source for the heightened interest in the advocacy for the possible elimination of anopheline-transmitted filariasis; however, a study has observed “facilitation” in *An. gambiae* s.s. and *An. arabiensis* but not in *An. melas* in Gambia or *An. merus* in Tanzania [4]. Additional health benefits of MDA targeting LF is the reduction in soil transmitted helminths and scabies [8].

A study in the Bongo district of northern Ghana [9] indicates a plausible “limitation” in *An. gambiae* s.l. and/or *An. funestus* in the transmission of the parasite contrary to other reports [4]. Results from a study in the Gomoa district of southern Ghana also indicated that although transmission potential by *An. funestus* has decreased significantly after mass chemotherapy with ivermectin and albendazole, there appears to be no change in *An. gambiae* s.s. in the area (Boakye DA, unpublished data). This suggests that probably not all anophelines exhibit facilitation in their transmission of LF. This work was therefore conducted to determine the roles of these two *Anopheles* species in the transmission of low level *W. bancrofti* human mf, since this information is fundamental to the success of GPELF.

## Methods

### Study sites

Nine LF endemic communities in the Gomoa district of Ghana (between Latitude 5° 24’ - 35’N and Longitude 0° 25’ - 36’W) were selected based on available data on the disease epidemiology in the population and vector species distribution [10-12]. These are Amanful, Ayesuano, Dago, Fawomanye, Hwida, Kyiren, Mampong, Obiri and Okyereko. The district lies in the coastal savannah zone of Ghana and is located 50 km west of Accra, the capital city of Ghana. Average annual rainfall ranges between 760 and 1000 mm, whilst mean annual temperature ranges between 26 and 30°C. The main occupations of the inhabitants are farming and fishing for those living near the shores of the Atlantic Ocean.

### Mass screening for microfilariae in the communities

This study was conducted from April to June 2004, the fourth year of MDA with ivermectin and albendazole in these communities. The areas also form part of an on-going annual longitudinal community-based intervention study. Human participation and the mosquito collection were done by cluster sampling method. Mass screening of the study population for mf was done by collecting 100 μl finger-pricked blood from each individual into heparinised capillary tubes and immediately mixing with 900 μl 3% acetic acid. Quantification of parasitaemia used the Sedgwick-Rafter counting chamber method with the compound microscope set at ×100 magnification [13].

### Mosquito collection, maintenance and dissection

After consenting to participate, twelve adult volunteers with varying mf levels slept under partially opened mosquito nets hung over beds in their rooms. At the mid-point of each collection hour, finger-pricked blood was taken and mf density estimated using the same procedure described above. Mosquitoes trapped in the nets were collected each hour from 21.00 hours to 06.00 hours on the next day using an aspirator. About half the number of mosquitoes collected were killed immediately and dissected for ingested mf. The remaining mosquitoes were fed on 10% sugar solution and maintained for up to 13 days in paper-cups at 26-28×C, relative humidity 70-80% and 12-hour photoperiod in the insectary [14]. Mosquitoes that died before day 13 were dissected for developing stages of *W. bancrofti*, whilst those that survived until the last day were dissected for the presence of infective stage L_3_ larvae of the parasite.

### PCR identification of *Anopheles* species

Molecular identifications of *An. gambiae, An. funestus* and *W. bancrofti* were conducted using already established methods [15-17]. For the vector species identification, genomic DNA was extracted from the carcasses of mosquitoes after homogenisation with sterile Konte’s plastic pestles in 100 μl bender buffer. The homogenate was then incubated at 65°C for 30 min, followed by the addition of 125 μl of phenol. The Centrifuge 5415 C (Eppendorf) was used in all spinning of samples, unless otherwise stated. The mixture was vortexed and spun at 14,000 rpm for 10 min. The supernatant was transferred into a fresh tube and 250 μl of pre-chilled absolute ethanol and 10 μl of 8 M potassium acetate were added. This was incubated at -40°C for an hour, spun at 10,000 rpm for 10 min and supernatant poured off. The pellet was then rinsed with 200 μl of 70% ethanol, spun at 10,000 rpm for 5 min, and supernatant poured off. The pellet was dried and re-dissolved in 50 μl TE + RNAse and then kept at 4°C until ready for PCR (Table 1). Each PCR reaction mixture of 25 μl contained 1× PCR buffer (Sigma, USA), 200 μM each of the four deoxyribonucleotide triphosphates, 10 μM each of the oligonucleotide primers (Table 1), and 0.125 units of *Taq* Polymerase enzyme (Sigma, USA). A microliter of the genomic DNA was used as template for the amplification reaction. *Anopheles gambiae* s.s. were further identified and differentiated into the M and S molecular forms by enzymatic restriction of the PCR product as described by Fanello *et al*. [18]. This was done by amplification of 1.3 kb rDNA followed by restriction fragment length polymorphism (RFLP) with restriction enzyme *Hha* I (Sigma-Aldrich, USA).

**Table 1:** Oligonucleotide primer sequences and PCR reaction conditions for species identification

**Primer for species ID**	**Sequence 5’** ⇒ **3’**	**PCR product size (bp)**	**PCR conditions**
***Anopheles gambiae *** **s.l. species**			
Universal primer	GTGTGCCCCTTCCTCGATGT	468	93°C 3’ followed by 35 cycles (93°C 30”; 50°C 30”; 72°C 1’); 93°C 30”; 50°C 30”; 72°C 10’
*Anopheles gambiae s.s.*	CTGGTTTGGTCGGCACGTTT	390	
*Anopheles merus/melax*	TGACCAACCCACTCCCTTGA	464	
*Anopheles arabiensis*	AAGTGTCCTTCTCCATCCTA	315	
*Anopheles quadrianulatus*	CAGACCAAGATGGTTAGTAT	153	
***Anopheles funestus *** **s.l. species**			
Universal primer	TGTGAACTGCAGGACACAT		30 cycles (94°C 30”; 40°C 30”; 72°C 30”); 72°C 10’
*Anopheles funestus* s.s.	GCATCGATGGGTTAATCATG	460	
*Anopheles vaneedeni*	TGTCGACTTGGTAGCCGAAC	555	
*Anopheles rivulorum*	CAAGCCGTTCGACCCTGATT	400	
*Anopheles parensis*	TGCGGTCCCAAGCTAGGTTC	235	
*Anopheles leesoni*	TACACGGGCGCCATGTAGTT	146	
***Wuchereria bancrofti***			
*NV-1*	CGTGATGGCATCAAAGTAGCG	188	94°C 3’; followed by 35 cycles (94°C 1’; 55°C 1’; 72°C 2’); 94°C 1’; 55°C 1’; 72°C 10”
*NV-2*	CCCTCACTTACCATAAGACAAC	188	

After gene amplification and digestion, the PCR products were electrophoresed separately in 2% agarose gel. The gel was prepared by adding TAE buffer to the powder, which was placed in a microwave oven (230 V, 50 Hz, 2660 W, 12.0A) for 1 minute to dissolve the solute, and then stained with 0.5 μg/ml Ethidium Bromide. For the electrophoresis, 8 μl of each sample was added to 1 μl of orange G (5X) gel loading dye after placing the solidified gel in 1X TAE buffer in a mini gel system (BIORAD USA). One hundred volts of electric current was passed through it for an hour and the gel photographed over a UV trans-illuminator (UPC, USA) at short wavelength using a Polaroid camera and film type 667 (Polaroid, USA). The sizes of the PCR products were estimated by comparison with the mobility of a 100 base pair molecular weight size marker (Sigma).

### Molecular identification of *Wuchereria bancrofti* larvae in mosquito vectors

After the carcass of infected mosquito was scrapped into 1.5 ml eppendorf tubes, DNeasy Tissue Kit (QIAGEN Inc., USA) was used in the extraction of the parasite’s genomic DNA from animal tissues following the manufacturer’s recommended protocol. After the DNA extraction, aliquots of 5 μl of the filarial DNA extract from the mosquitoes were used as templates for the amplification reaction. The PCR assay was performed using two published specific oligonucleotide primers, NV-1 and NV-2 [17]. The PCR products were electrophoresed in 2% agarose gel as described in the previous section.

### Molecular identification of *Wuchereria bancrofti* microfilariae in human blood

Microfilariae (mf) in human blood samples that were preserved in 3% acetic acid were also characterised after extracting the genomic DNA using the same kit described above. Infected blood samples were amplified and identified using the same procedure described in previous sections.

### Ethical considerations

For the yearly MDA and mass screening for mf prevalence in the communities, oral informed consent was sought from all participants. Subsequently, written consent was obtained from each volunteer who slept under bed nets after the study purpose, procedures, entry and exit criteria were explained to them. All volunteers and the entire community members received that year’s round of MDA immediately after the blood sample collection. The Institutional Review Board of Noguchi Memorial Institute for Medical Research approved the study.

### Statistical analysis

Data were entered into Microsoft Access and analysed for the vector competency of *Anopheles* spp. in supporting the development of mf to the infective stage larvae. The same software was used to calculate the geometric mean intensity on mf in the human population. One-way analysis of variance (ANOVA) was used to test for the significance of age- and gender-specific variations between the human population and mf, with *p* value set at 0.05.

## Results

### Human microfilariae load in the communities after four rounds of MDA

The overall prevalence of mf in the study communities (*N* = 1083) was 1.6%; mf prevalence among males and females (2.05% and 1.10% respectively) was not significantly different (*p* = 0.39). The mf levels ranged from 0 to 59/100 μl blood with geometric mean intensity of 1.1 mf/ml of blood (Table 2). There was no significant variation in mf intensity and age-group (*p* = 0.40); likewise no significant difference between mf intensity and gender of participants (*p* = 0.91) (Table 2). Four out of the nine communities namely Ayesuano, Dago, Hwida and Okyereko recorded positive cases, with Okyereko having the highest number of cases (Table 3). Among the positive cases, Okyereko recorded 155.6 mf/ml of blood whilst Dago recorded 15.3 mf/ml of blood.

**Table 2:** Prevalence of mf and the geometric mean intensity in the study area

**Age group (yrs)**	**Individuals examined**			**mf positive individual (%)**			*mf geometric mean intensity			
	**Female**	**Male**	**Total**	**Female**	**Male**	**Total**	**Female**	**Male**	**Total**	**mf positives only**
1-14	242	252	494	2 (0.36)	3 (0.56)	5 (0.46)	1.05	1.05	1.05	127.85
15-24	114	137	251	0	5 (0.93)	5 (0.46)	0	1.18	1.05	86.40
25-34	57	44	101	0	0	0	0	0	0	0
35-44	42	30	72	1 (0.18)	0	1 (0.09)	1.10	0	1.06	51
45+	92	73	165	3 (0.55)	3 (0.56)	6 (0.55)	1.10	1.23	1.16	53.66
**All**	**547**	**536**	**1083**	**6 (1.10)**	**11 (2.05)**	**17 (1.57)**	**1.05**	**1.10**	**1.07**	**79.45**

*Antilog [Σ log (x + 1)/ n], where x is the number of mf per ml of blood in mf individuals and n is the number of people examined [9].

**Table 3:** **Blood sampling results showing number of people infected with microfilaria of *Wuchereria bancrofti *** **in the year 2004 (four years of MDA)**

**Study communities**	**Number examined**	**Number positive (mf density/ml of blood)**	**Geometric mean intensity (mf/ml)**
Amanful	61	0	0
Ayesuano	69	1 (4)	1.0
Dago	228	4 (1 – 15.3)	1.0
Fawomanyo	66	0	0
Hwida	88	2 (5 – 21)	1.0
Kyiren	161	0	0
Mampong	63	0	0
Obiri	70	0	0
Okyereko	277	10 (1 – 155.6)	1.2

### Mosquito species composition and entomological indices

**Figure 1:**
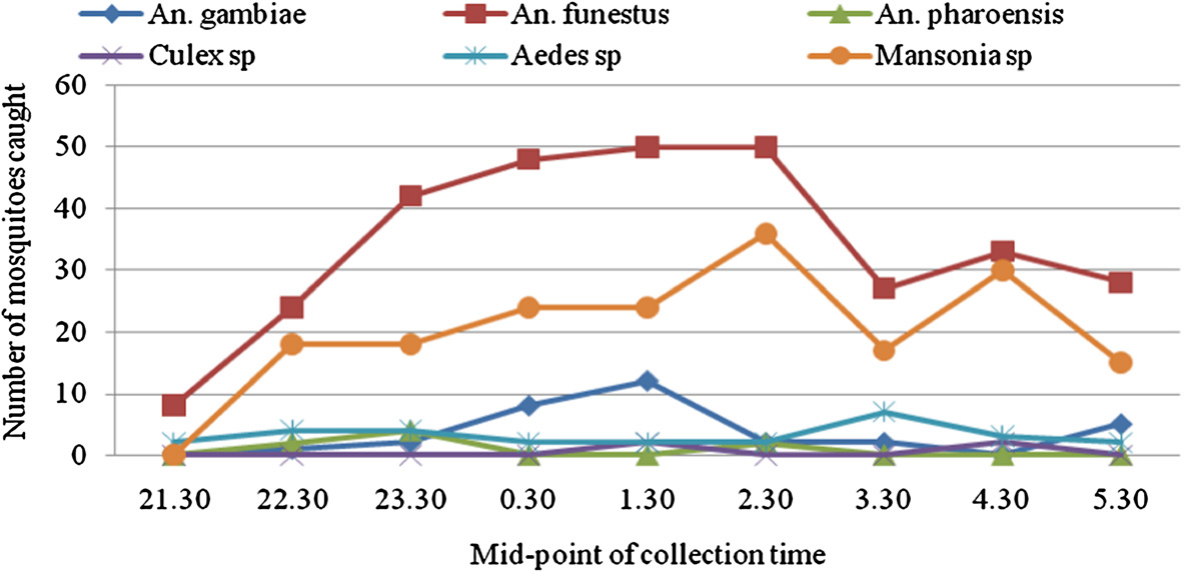
Hourly distribution of the mosquito species that were caught from the bed nets under which volunteers were sleeping.

**Figure 2:**
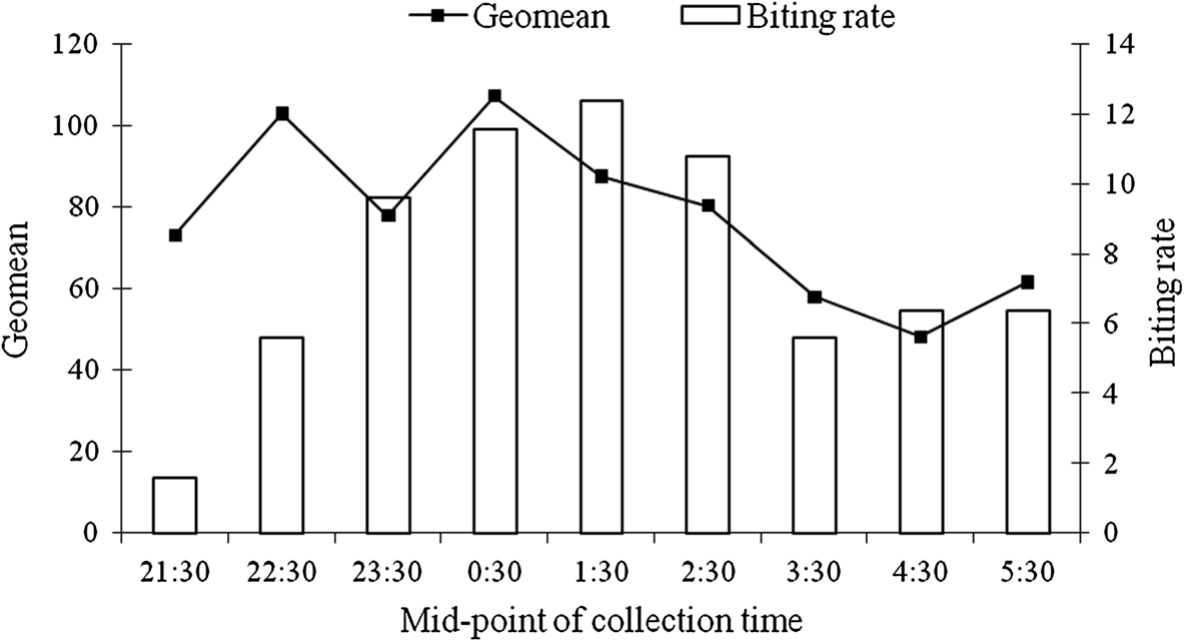
**Biting rate of *Anopheles *** **species and the geometric mean intensity (Geomean) of mf that were observed during the night of sample collection.**

The 564 mosquitoes collected consisted of 350 (62.1%) *Anopheles*, 182 (32.3%) *Mansonia*, 28 (5%) *Aedes* and 4 (0.7%) *Culex* species, (Figure 1). The *Anopheles* species comprised of 310 (88.6%) *An. funestus,* 32 (9.1%) *An. gambiae* and 8 (2.3%) *An. Pharoensis,* (Figure 1). The hourly biting rates of *An. gambiae* and *An. funestus* were 6.4 and 62 bites/person/night respectively (Table 4). Of the mosquito species collected, 192 (34%) were engorged with blood-meals (Table 5). Whereas 6/350 (1.7%) of the *Anopheles* and 6/182 (3.3%) of the *Mansonia* species were found with the mf (L_1_ stage) of *W. bancrofti*, there was no recovery of L_3_ or L_2_ stage larvae after 12 days of maintenance. While each of the infected *An. gambiae* had an average of one mf, each *An. funestus* had an average of eight mf when killed immediately after collection (Table 5). The mf load in the peripheral blood and biting rates of *Anopheles* mosquitoes peaked concurrently between 0.30 and 2.30 hours (Figure 2).

**Table 4:** Entomological indices of the various mosquito species that were caught during the study

**Mosquito species**	**Entomological indices (%)**					
	**Biting rate**^**a**^	**Infection rate**^**b**^	**Infectivity rate**^**c**^	**Intensity of infection**^**d**^	**Survival rate**^**e**^	**Vector efficiency**^**f**^
*An. gambiae*	6.4	0.13	0	0	0.50	0
*An. funestus*	62.0	0.03	0	0	0.47	0
*An. pharoensis*	1.6	0	0	0	1.0	0
*Culex* sp	0.8	0	0	0	0	0
*Aedes* sp	5.6	0	0	0	5.0	0
*Mansonia* sp	36.4	0.07	0	0	0.12	0

^a^Number of mosquitoes caught/ number of collectors x number of captures (bites/ person/ night); ^b^Number of mosquitoes infected/ number of mosquitoes dissected; ^c^L_3_ in the head and proboscis/ number of surviving mosquitoes; ^d^Number of mosquitoes with L_3_ in the head and proboscis/ number of mosquitoes with L_3_; ^e^Number of surviving mosquitoes at the end of study/ number of engorged mosquitoes. ^f^Number of L_3_ x 100/ number of mf ingested among dissected mosquitoes [19-21].

**Table 5:** Number of mosquitoes caught and examined before and after maintenance in the laboratory

**Mosquito species**	**No: of mosquitoes**		**No: of mosquitoes examined immediately after collection**		**No: of mosquitoes examined from days 1-8 of maintenance**		**No: of mosquitoes examined from days 9-13 of maintenance**	
	**Caught**	**Engorged with blood**	**Dissected**	**Infected (no: mf)**	**Dissected**	**Infected (no: mf)**	**Dissected**	**Infected**
*An. gambiae*	32	12	16	2 (2)	10	2 (3)	6	0
*An. funestus*	310	98	155	4 (33)	109	4 (4)	46	0
*An. pharoensis*	8	2	4	0 (0)	2	0	2	0
*Culex* sp.	4	2	2	0 (0)	2	0	0	0
*Aedes* sp.	28	2	14	0 (0)	4	0	10	0
*Mansonia* sp.	182	76	90	6 (12)	82	7 (7)	9	0
**Total**	**564**	**192**	**281**	**12 (47)**	**209**	**13 (14)**	**73**	**0**

All mf found in mosquitoes were of L_1_ stage, neither L_2_ nor L_3_ stages were found. All mosquitoes caught were dissected latest by end of the maintenance (13 days).

### PCR identification of *Anopheles* mosquitoes and *Wuchereria bancrofti*

Of the 32 *An. gambiae* s.l. collected, 30 were identified as *An. gambiae* s.s. as they showed the expected diagnostic band size of 390 base pairs. After restriction enzyme treatment with *Hha* I, M forms remained a single band of 390 bp since there was no digestion. S forms on the other hand resulted in two bands of 110 and 280 bp. Of the *An. gambiae* s.s digested, 21 (70%) were M forms with 9 (30%) S molecular forms. Among the 310 *An. funestus* s.l. collected, 286 were identified by PCR; of which 267 (86%) were *An. funestus* s.s. with diagnostic band sizes of 460 base pairs, and 19 (6%) were *An. Leesoni* with band sizes of 146 base pairs. The presence of *W. bancrofti* in 20 infected mosquitoes and 10 human blood samples were confirmed at 188 bp.

## Discussion

An LF-endemic community is said to have low mf density when the density of circulating mf is less than 200 mf per ml of blood, an amount which cannot be detected in a significant number of instances when commonly used blood sampling techniques are employed [4]. Nonetheless, this depends on variables such as volume of blood examined, source of blood sampled (venous or capillary) and method of mf detection that is employed. In this study, 100 μl of finger-prick blood was used, which is an appreciable amount of blood compared to the popular technique for mf detection in routine public health practice of 20 μl finger-prick blood [4]. As such it could be inferred that the mean mf intensity of 1.07 and 79.45 mf per ml of capillary blood in the entire study communities and mf positive individuals respectively were really low in the studied area. This may be due to the 66.6% overall coverage rate in MDA with ivermectin and albendazole for 4 years leading to a reduction in mf densities among the inhabitants (Boakye DA, unpublished data). Evidence from Okyereko supports this view that MDA has been effective; in this study period, 155.6 mf per ml of blood were recorded among mf positive individuals, hitherto the commencement of MDA, as high as 819 mf/ml of blood were recorded among this group [12].

Various observations have been made regarding mf prevalence and intensities in study populations [9,11,12,22]. These could be attributed in part to the occupational activities of inhabitants of the study areas as well as the biting pattern of the local anopheline vectors, which are presently known to be the main vectors of LF in Ghana [10-12]. Studies on the relationship between mf density in blood meals and the percentage of *Anopheles* mosquitoes that ingest mf have not provided consistent results [3,9,22-25]. Southgate and Bryan [26] showed that although many of the mf ingested by *Anopheles* vectors are damaged by the mosquito’s foregut armature, the proportion of mf destroyed does not depend on the number of mf ingested and varies between members of the *An. gambiae* complex and *An. funestus*. It is therefore not proper to extend findings from a given area to the other even for the same species. Additionally, other anatomical structures and immune factors other than the foregut armature could be modulating mf density following ingestion. Further studies are thus required to provide more understanding into the vector-parasite relationships.

Indeed the significance of distinctly different host-parasite relationships lies in the importance of low-density mf in sustaining transmission in various endemic areas with different genera of mosquito vectors [4]. As hypothesised, the theory of “limitation” allows transmission to occur and build-up when most infected human hosts have low mf densities, whereas situations of well marked “facilitation” will give rise to transmission thresholds below which transmission will ultimately cease leading to parasite elimination from the human population. However, such predictions of parasite extinction or parasite resurgence can only be made with confidence when characteristics of the local vector-mf relationship are well understood [7].

As part of our study, we described the circadian pattern of mf periodicity in southern Ghana. Based on hourly examination of twelve volunteers for nine hours, we observed that mf concentration in peripheral blood followed a wave-like concentration peaking around 01.00 hours, which was similar to other findings [12,27,28]. This interesting behavioural pattern of mf is said to be the parasite’s response to oxygen tension, which is high in peripheral circulation at night due to the low human activity at this time of the day [29,30].

In their study, Dzodzomenyo M. *et al*. [12] observed *An. funestus* to be the most abundant mosquito species in the early dry season while *An. gambiae* was predominant in the wet season. Our study was conducted in March, which is the peak of the dry season in Ghana and thus may contribute to the low number of *An. gambiae* that were captured. Studies show that the M and S forms of *Anopheles gambiae* s.s. do occur in sympatry in southern Ghana [31]. Our study revealed that most of the *Anopheles gambiae* s.s. were M form, which has a remarkable ecological flexibility and is known to prevail in inundated areas where dry season breeding opportunities exist [10]. Further studies could look at the role of these molecular forms of *Anopheles gambiae* s.s. in transmission of *W. bancrofti* following MDA.

## Conclusion

After 4 rounds of mass drug administration, parasitaemia was brought to a low level in the study communities. Low levels of circulating microfilariae in the inhabitants might have contributed to the no recovery of infective stage larvae of *W. bancrofti* in *An. funestus* s.l. as well as M and S forms of *An. gambiae*. Although the mosquito numbers were low, a further study is recommended to ascertain this observation.

## Competing interest

The authors declare that they have no competing interests.

## Authors’ contributions

All authors contributed significantly to this study. DAB and MDW conceived the idea and design of the study. BKB, FAA and JO carried out the field and laboratory studies. BKB prepared the manuscript, while all authors read and approved the final manuscript.

## References

[B1] Michael E Bundy DA Grenfell BT Re-assessing the global prevalence and distribution of lymphatic filariasis Parasitol 1996 112 409 428 10.1017/S0031182000066646 8935952

[B2] Crans WJ Experimental infection of *Anopheles gambiae* and *Culex quinquefasciatus pipiens fatigans* with *Wuchereria bancrofti* in coastal East Africa J Med Entomol 1973 10 189 193 4707754 10.1093/jmedent/10.2.189

[B3] McGreevy PB Kolstrup N Tao J McGreevy MM Marshall TF Ingestion and development of *Wuchereria bancrofti* in *Culex quinquefasciatus, Anopheles gambiae* and *Aedes aegypti* after feeding on humans with varying densities of microfilariae in Tanzania Trans R Soc Trop Med Hyg 1982 76 288 296 10.1016/0035-9203(82)90170-5 6126022

[B4] Southgate BA The significance of low density microfilareamia in the transmission of lymphatic filarial parasites J Trop Med Hyg 1992 95 79 86 1348543

[B5] Zhang SQ Zhang QJ Cheng F Wang LL Pen GP Threshold of transmission of *Brugia malayi* by *Anopheles sinensis* J Trop Med Hyg 1991 94 245 250 1880826

[B6] Yamey G Global alliance launches plan to eliminate lymphatic filariasis BMJ 2000 320 269 10.1136/bmj.320.7230.269 10650014 PMC1117478

[B7] Webber RH Eradication of *Wuchereria bancrofti* through vector control Trans R Soc Trop Med Hyg 1991 73 722 724 395730 10.1016/0035-9203(79)90031-2

[B8] Mohammed KA Deb RM Stanton MC Molyneux DH Soil transmitted helminths and scabies in Zanzibar, Tanzania following mass drug administration for lymphatic filariasis – a rapid assessment methodology to assess impact Parasit Vectors 2012 5 299 10.1186/1756-3305-5-299 23259465 PMC3543323

[B9] Boakye DA Wilson MD Appawu MA Gyapong J Vector competence for *Wuchereria bancrofti* of the *Anopheles* populations in the Bongo District of Ghana Ann Trop Med Parasitol 2004 98 501 508 10.1179/000349804225003514 15257800

[B10] Appawu MA Baffoe-Wilmot A Afari EA Nkrumah FK Petrarca V Species composition and inversion polymorphism of the *Anopheles gambiae* complex in some sites of Ghana, West Africa Acta Trop 1994 56 15 23 10.1016/0001-706X(94)90036-1 8203292

[B11] Dunyo SK Appawu M Nkrumah FK Baffoe-Wilmot A Pedersen EM Simonsen PE Lymphatic filariasis on the coast of Ghana Trans R Soc Trop Med Hyg 1996 90 634 638 10.1016/S0035-9203(96)90414-9 9015499

[B12] Dzodzomenyo M Dunyo SK Ahorlu CK Coker WZ Appawu MA Pedersen EM Simonsen PE Bancroftian filariasis in an irrigated project community in southern Ghana Trop Med Int Health 1999 4 13 18 10.1046/j.1365-3156.1999.00354.x 10203168

[B13] McMahon JE Marshall TF d C Vaughan JP Abaru DE Bancroftian filariasis: a comparison of microfilariae counting techniques using counting chamber, standard slide and membrane (Nucleopore) filtration Ann Trop Med Parasitol 1979 73 457 464 393190 10.1080/00034983.1979.11687285

[B14] Janousek TE Lowrie RC Jr Vector competency of *Culex quinquefasciatus* (Haitian strain) following infection with *Wuchereria bancrofti* Trans R Soc Trop Med Hyg 1989 83 679 680 10.1016/0035-9203(89)90395-7 2694503

[B15] Collins FH Mendez MA Rasmussen MO Mehaffey PC Besansky NJ Finnerty V A ribosomal RNA gene probe differentiates member species of the *Anopheles gambiae* complex Am J Trop Med Hyg 1987 37 37 41 2886070 10.4269/ajtmh.1987.37.37

[B16] Scott JA Brogdon WG Collins FH Identification of single specimens of the *Anopheles gambiae* complex by the polymerase chain reaction Am J Trop Med Hyg 1993 49 520 529 8214283 10.4269/ajtmh.1993.49.520

[B17] Ramzy RM Farid HA Kamal IH Ghada HI Zakariah SM Rifky F Weil GJ Williams SA Gad AM A polymerase chain reaction–based assay for detection of *Wuchereria bancrofti* in human blood and *Culex pipiens* Trans R Soc Trop Med Hyg 1997 91 156 160 10.1016/S0035-9203(97)90205-4 9196756

[B18] Fanello C Santolamazza F della Torre A Simultaneous identification of species and molecular forms of the *Anapheles gambiae* complex by PCR-RFLP Med Vet Entomol 2002 16 461 464 10.1046/j.1365-2915.2002.00393.x 12510902

[B19] Brito AC Williams P Fontes G Rocha EMM A comparison of two Brazilian populations of *Culex quinquefasciatus* (Say, 1823) from endemic and non-endemic areas to infection with *Wuchereria bancrofti* (Cobbold, 1877) Mem Inst Oswaldo Cruz 1997 92 33 36 10.1590/S0074-02761997000100007 9302411

[B20] Kartman L Suggestions concerning an index of experimental filarial infection in mosquitoes Am J Trop Med Hyg 1954 3 329 337 13138835 10.4269/ajtmh.1954.3.329

[B21] Ramachandran CP A guide to methods and techniques in Filariasis Investigations Filar Res Off Inst Med Res, Kuala Lumpur 1970 39pp

[B22] Coulibaly YI Dembele B Diallo AA Kristensen S Konate S Dolo H Dicko I Sangare MB Keita F Boatin BA Traore AK Nutman TB Klion AD Touré YT Traore SF *Wuchereria bancrofti* transmission pattern in southern Mali prior to and following the institution of mass drug administration Parasit Vectors 2013 6 247 10.1186/1756-3305-6-247 23981378 PMC3765776

[B23] Bryan JH McMahon P Barnes A Factors affecting transmission of *Wuchereria bancrofti* by anopheline mosquitoes. 3. Uptake and damage to ingested microfilariae by *An. gambiae*, *An. arabiensis*, *An. merus* and *An. funestus* in East Africa Trans R Soc Trop Med Hyg 1990 84 265 268 10.1016/0035-9203(90)90281-I 2202106

[B24] Bryan JH Southgate BA Factors affecting transmission of *Wuchereria bancrofti* by anopheles mosquitoes. 1. Uptake of microfilariae Trans R Soc Trop Med Hyg 1988 82 128 137 10.1016/0035-9203(88)90286-6 3051542

[B25] Bryan JH Southgate BA Factors affecting transmission of *Wuchereria bancrofti* by anopheles mosquitoes. 2. Damage to ingested microfilariae by mosquito foregut armatures and development of filarial larvae in mosquitoes Trans R Soc Trop Med Hyg 1988 82 138 145 10.1016/0035-9203(88)90288-X 3051543

[B26] Southgate BA Bryan JH Factors affecting transmission of *Wuchereria bancrofti* by anopheline mosquitoes. 4. Facilitation, limitation, proportionality and their epidemiological significance Trans R Soc Trop Med Hyg 1992 86 523 530 10.1016/0035-9203(92)90096-U 1475823

[B27] Tanaka H Periodicity of microfilariae of human filariasis analysed by a trigonometric method (Aikat and Das) Jpn J Exp Med 1981 51 97 103 7024589

[B28] Gatika SM Fugimaki Y Njuguna MN Gachihi GS Mbugua JM The microfilarial periodicity pattern of *Wuchereria bancrofti* in Kenya J Trop Med Hyg 1994 97 60 64 8107176

[B29] Hawking F Pattanayak S Sharma HL The periodicity of microfilariae XI. The effect of body temperature and other stimuli upon the cycles of *Wuchereria bancrofti, Brugia malayi, B. ceylonensis* and *Dirofilaria repens* Trans R Soc Trop Med Hyg 1966 60 496 513 10.1016/0035-9203(66)90275-6 5955121

[B30] Denham DA McGreevy PB Brugian filariasis: epidemiological and experimental studies Adv Parasitol 1977 15 243 309 17276 10.1016/s0065-308x(08)60530-8

[B31] Yawson AE McCall PJ Wilson MD Donnelly MJ Species abundance and insecticide resistance of *Anopheles gambiae* in selected areas of Ghana and Burkina Faso Med Vet Entomol 2004 18 372 377 10.1111/j.0269-283X.2004.00519.x 15642004

